# (Dis)Agreement in Siblings’ Perceptions of Parents’ Dementia Symptoms: Impact on Caregiver Burden

**DOI:** 10.1093/geroni/igaf122.2073

**Published:** 2025-12-31

**Authors:** Hanamori Skoblow, Megan Gilligan, Destiny Ogle, Audrey Deyoung

**Affiliations:** University of Missouri, Columbia, Missouri, United States; University of Missouri, Columbia, Missouri, United States; Purdue University, West Lafayette, Indiana, United States; University of Missouri, Columbia, Missouri, United States

## Abstract

More severe behavioral and psychological symptoms of dementia (BPSD) are associated with higher caregiver burden. Typically, BPSD is measured using reports from one caregiver although disagreements between multiple caregivers may exacerbate the challenges of care. However, the consequences of caregivers’ disagreements regarding BPSD symptoms remain unknown. Therefore, we examined whether adult child caregivers’ (dis)agreements on total BPSD symptoms and subscales for depression, disturbance, and memory were associated with sibling reports of caregiver burden. We used data from 91 sibling dyads (mean age = 57.30, SD = 9.32) who were caring for a parent with dementia (mean age = 82.30, SD = 7.74). Actor-partner interdependence models revealed that sibling dyads who reported minor disagreements (within 1 SD) regarding their parent’s total BPSD symptoms reported higher caregiver burden compared to siblings who were in agreement about their parent’s symptoms (b = 2.67). This effect was primarily driven by disagreements on the parent’s depressive symptoms, where major disagreements (>1 SD) were associated with heightened caregiver burden (b = 1.81), relative to siblings who agreed on their parent’s symptoms. In contrast, disagreements on the parents’ disturbance and memory symptoms were not linked to sibling caregiver burden. These findings suggest that when siblings’ perceptions of their parent’s depressive symptoms are aligned, they report lower caregiver burden. This research has implications for health and long-term care providers, whose contact regarding the parent’s symptoms may be limited to only one caregiver. Ensuring consistency and alignment across the care network may help promote better caregiver well-being.

